# Optical Logic Gates Based on Z-Shaped Silicon Waveguides at 1.55 μm

**DOI:** 10.3390/mi14061266

**Published:** 2023-06-18

**Authors:** Amer Kotb, Kyriakos E. Zoiros, Antonios Hatziefremidis, Chunlei Guo

**Affiliations:** 1 GPL Photonics Laboratory, State Key Laboratory of Luminescence and Applications, Changchun Institute of Optics, Fine Mechanics and Physics, Chinese Academy of Sciences, Changchun 130033, China; 2 Department of Physics, Faculty of Science, University of Fayoum, Fayoum 63514, Egypt; 3 Lightwave Communications Research Group, Department of Electrical and Computer Engineering, School of Engineering, Democritus University of Thrace, 67100 Xanthi, Greece; 4 Department of Aerospace Science and Technology, National Kapodistrian University of Athens, 34400 Psahna Evias, Greece; 5 The Institute of Optics, University of Rochester, Rochester, NY 14627, USA

**Keywords:** optical logic gates, silicon waveguide, Z shape, contrast ratio

## Abstract

In the last ten years, silicon photonics has made considerable strides in terms of device functionality, performance, and circuit integration for a variety of practical uses, including communication, sensing, and information processing. In this work, we theoretically demonstrate a complete family of all-optical logic gates (AOLGs), including XOR, AND, OR, NOT, NOR, NAND, and XNOR, through finite-difference-time-domain simulations using compact silicon-on-silica optical waveguides that operate at 1.55 μm. Three slots, grouped in the shape of the letter Z, make up the suggested waveguide. The function of the target logic gates is based on constructive and destructive interferences that result from the phase difference experienced by the launched input optical beams. These gates are evaluated against the contrast ratio (CR) by investigating the impact of key operating parameters on this metric. The obtained results indicate that the proposed waveguide can realize AOLGs at a higher speed of 120 Gb/s with better CRs compared to other reported designs. This suggests that AOLGs could be realized in an affordable manner and with improved outcomes to enable the satisfaction of the current and future requirements of lightwave circuits and systems that critically rely on AOLGs as core building elements.

## 1. Introduction

By merely expanding the quantity of discrete optical channels, the extreme complexity of next-generation high-capacity data transmission systems cannot be solved. The integration density of optoelectronic devices can be greatly increased while keeping cost and energy consumption low owing to silicon photonics. The silicon-on-insulator (SOI) platform, a production method in which a thin silicon layer is placed on top of an insulator substrate formed of silica (i.e., SiO_2_), uses silicon as its primary raw material. Due to waveguiding silicon’s high refractive index (n_silicon_ = 3.48) in comparison to air (n_air_ = 1) or the silica cladding layer (n_silica_ = 1.44), the strong optical guiding is ensured for all signals around the typical near-infrared wavelength of 1.55 μm. There are many excellent reasons why the SOI platform has evolved into silicon photonics. For instance, silicon is broadly accessible and compatible with advanced CMOS technology, making it possible to produce structures with sizes as small as 10 nm at a reasonable price [1,2,3,4,5,6]. Due to silicon’s strong optical confinement, which allows for bending waveguide radii of only a few micrometers and functional waveguide elements of just ten to a few hundred micrometers, incredibly compact optical devices can be created [7]. In contrast, all-optical logic gates (AOLGs) overcome the disadvantages of their electronic counterparts, namely the low bandwidth and slow data transit speed, thus enabling more effective data processing. AOLGs have recently been realized using a variety of waveguide configurations [8,9,10,11,12,13,14,15,16,17,18,19,20,21,22,23,24,25,26,27,28,29]. In contrast to the basic waveguide suggested in this research, which can execute seven logic gates concurrently, the majority of these earlier designs have utilized photonic crystals (PCs) to execute only one or, at most, two logic gates [9,10,11,12,13,14,15,16,17]. Additionally, as opposed to silicon and silica that are proposed to be employed in the present design, other documented initiatives have utilized more affordable noble metals [23,24,25,26,27]. In line with earlier efforts [8,9,10,11,12,13,14,15,16,17,18,19,20,21,22,23,24,25,26,27,28,29], in this paper, we simulate a full family of AOLGs, including XOR, AND, OR, NOT, NOR, NAND, and XNOR, using Z-shaped silicon-on-silica waveguides at 1.55 μm telecommunications wavelength. Three slots that are arranged to form the letter Z make up the proposed waveguide. Based on the idea of constructive interference (CI) and destructive interference (DI) brought on by the phase discrepancies experienced by the launched input optical beams, these logic gates operate. In the FDTD simulations carried out in the commercially available Lumerical software [30], to evaluate and show the behavior of the suggested logic gates, a convolutional completely matched layer is employed as an absorbing boundary condition [22]. The performance of the proposed operations is evaluated against the contrast ratio (CR) metric. The simulation results reveal that owing to the devised waveguide-based structure, the target AOLGs can be executed with improved performance at a faster rate than other design counterparts described in the literature [8,12,13,14,19,22,24,25,26,27], which is technologically feasible. To this end, these AOLGs can serve as the key modules in fundamental- and system-oriented-level modern applications.

## 2. Waveguide Structure

**Figure 1:**
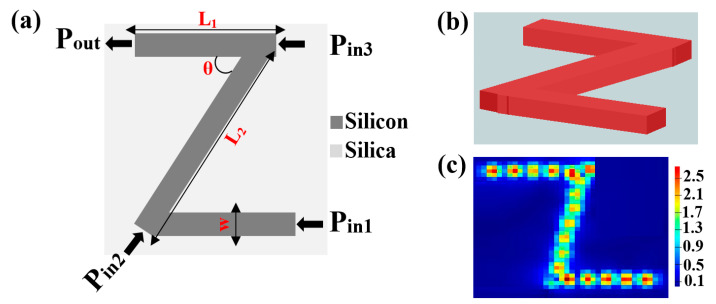
(**a**) Schematic illustration, (**b**) 3D FDTD view, and (**c**) field intensity distributions of the Z-shaped silicon waveguide.

The proposed waveguide, which has a silicon core and a silica substrate as the cladding, has three identical slots organized in the shape of the letter Z. The Z-shaped silicon waveguide is illustrated schematically in Figure 1, together with the 3D FDTD view and the corresponding field intensity distributions.

The three input ports of the waveguide are excited by a transverse magnetic mode polarized electromagnetic pulse at 1.55 μm. The simulation results are recorded by the FDTD monitors. The output spectral transmission (T) is$\;T=I_{\mathrm{out}}/I_{\mathrm{in}}$, where I_out_ is the intensity at the output port (P_out_) and I_in_ = I_1_ + I_2_ + I_3_ is the sum of the intensities at three input ports [22,27]. The normalized threshold transmission (T_th_), which denotes the minimal normalized power necessary to produce T, is initially chosen to have a value of 0.12. When T > T_th_, P_out_ produces ‘1’, while when T < T_th_, P_out_ produces a ‘0’. To optimize T, the input beams must meet specific phase-matching conditions [31,32]. DI scatters the beams when the waveguide’s and input beams’ phases are out of phase, producing an output of ‘0’. A crucial statistic for describing logic functions is the CR, which is defined as $\mathrm{CR}\left(\mathrm{dB}\right)=10\;\ln\left[P_{\mathrm{mean}}^{1}/P_{\mathrm{mean}}^{0}\right]$, where${\;P}_{\mathrm{mean}}^{1}\;$and $P_{\mathrm{mean}}^{0}$ are the mean peak powers of logic ‘1’ and ‘0’, respectively [22]. Table 1 contains the default simulation parameters. To obtain a higher CR, the FDTD simulations have been iteratively run to optimize these parameters.

## 3. Waveguide Performance

**Figure 2:**
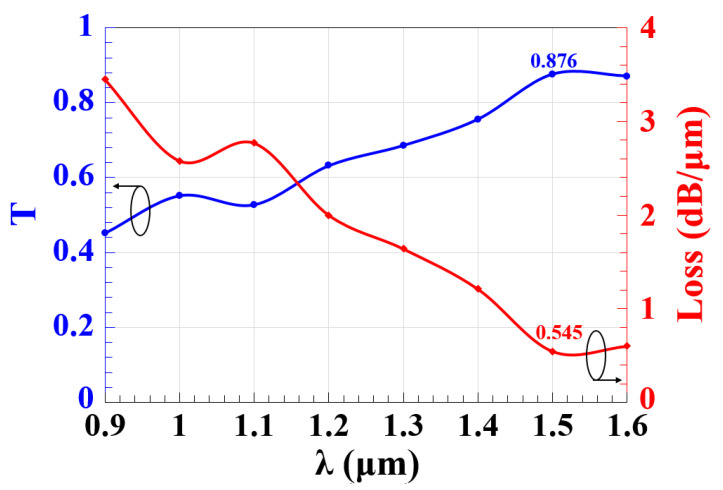
T and loss versus λ employing Z-shaped silicon waveguide.

Figure 2 illustrates T and the loss of the Z-shaped silicon waveguide as functions of λ, presuming that all incident beams are launched at the three input ports with an equal phase of 180°. Using the proposed waveguide, a high T of 0.876 and a low loss of 0.575 dB/μm are attained at 1.55 μm. These insignificant propagation losses are caused by scattering at the slots’ edges and material absorption. By elaborating more on this figure, it becomes clear that this waveguide operates with high T and low loss over 1.3–1.6 μm.

**Figure 3:**
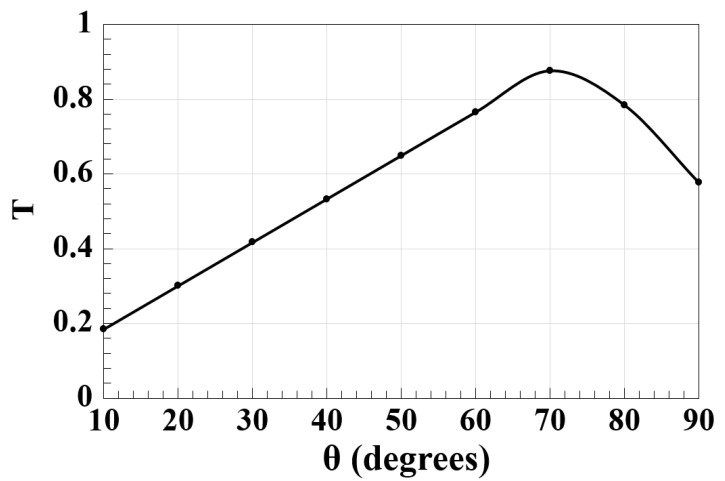
T versus angle between slots (θ) employing Z-shaped silicon waveguide at 1.55 μm.

The performance of the suggested waveguide is crucially influenced by the angle between slots (i.e., θ). As a consequence, using the suggested Z-shaped silicon waveguide, Figure 3 simulates the effect of this parameter on T at 1.55 μm. The highest T of 0.876 occurs at θ = 70°, making the latter the best value to use throughout simulations. By expanding on this figure, it can also be seen that by changing the value of θ, the amount of light scattering and absorption inside the materials is increased, which raises losses.

**Figure 4:**
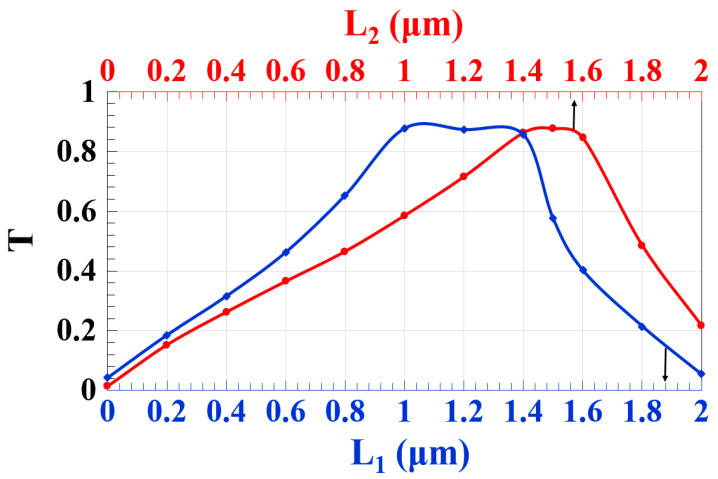
T versus length of the short slot (L_1_) and length of the long slot (L_2_) employing Z-shaped silicon waveguide at 1.55 μm.

The waveguide performance is significantly influenced by the shape of its arms. Therefore, T as a function of the length of the short slot (L_1_) and the length of the long slot (L_2_) at 1.55 μm is depicted in Figure 4. This figure demonstrates that the proposed Z-shaped silicon waveguide produces high T over the whole ranges of L_1_ and L_2_, i.e., L_1_ = 0.8–1.5 μm and L_2_ = 1.0–1.6 μm. This implies that the suggested design is practicable, especially in light of the accessibility of 3D femtosecond laser direct writing technology [33,34,35,36,37,38] and lithographical fabrication techniques [23,39,40,41].

**Figure 5:**
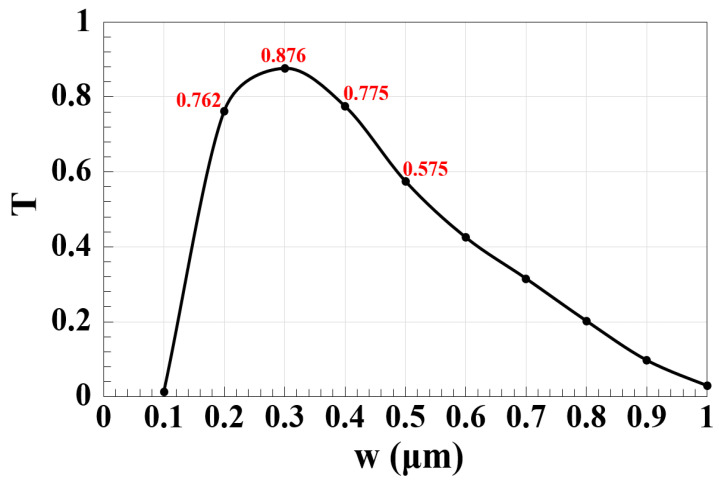
T versus width of the slot (w) employing Z-shaped silicon waveguide at 1.55 μm.

We examined the waveguide performance on the slot width (w) at 1.55 μm to obtain more accurate findings, as shown in Figure 5. It is clear from this figure that the Z-shaped silicon waveguide achieves high T for a wide range of w, i.e., 0.2–0.5 μm. With the advancement of nanofabrication techniques, this finding also shows that this design might be put to use and turned into a functional prototype [23,33,34,35,36,37,38,39,40,41].

The Nyquist formula, which is defined as$\;2B\log_{2}\left[M\right]$, where M is the total number of signal levels and B is the optical bandwidth, determines the speed of a transmission system [42]. In this formula, $B=(c/\lambda^{2})\Delta \lambda ,$ where *c* is the light speed in a vacuum, *λ* = 1.55 μm is the optical carrier wavelength, and Δ*λ* is the signal spectral width [22]. This indicates that for our case, where B = 30 GHz and M = 4 (i.e., 00, 01, 10, and 11), the employed waveguide operates at a high speed of 120 Gb/s.

## 4. XOR, AND, OR

The XOR, AND, and OR logic gates are implemented by injecting a clock beam (Clk) into P_in1_, while the other two input beams are fed into P_in2_ and P_in3_ (see Figure 1). The Clk (all ‘1’s) is required to induce interference, which can be either constructive or destructive by establishing a reference phase difference between the input beams. CI happens when all input beams are launched at the same phase (i.e., Φ_Clk_ = Φ_2_ = Φ_3_ = 180°) where the input beams interact in such a way that they are aligned, leading to a ‘1’ output. In contrast, DI occurs when these beams are launched at various phases (i.e., Φ_Clk_ = 180°, Φ_2_ = 0°, and Φ_3_ = 90°) and cancel each other out, leading to a ‘0’ output.

### 4.1. XOR

**Figure 6:**
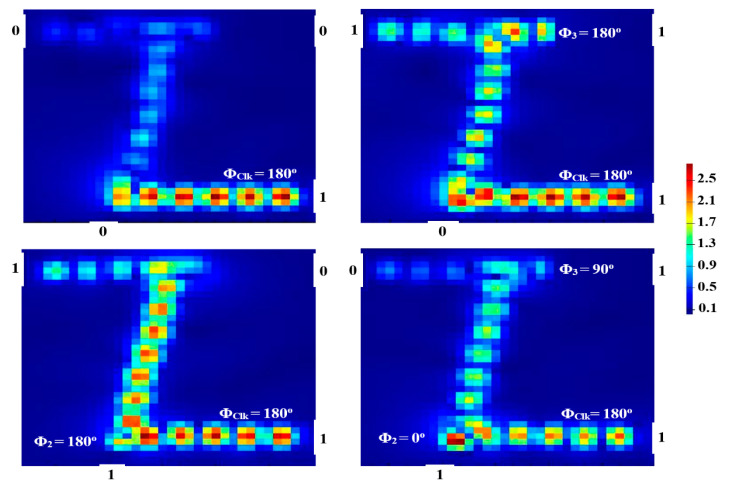
XOR field intensity distributions propagating through Z-shaped silicon waveguide at 1.55 μm.

P_out_ produces ‘1’ (i.e., T > T_th_) as a result of the CI between the input beams when the (01, 10) combination of the latter is injected along with the Clk at the same phase (i.e., Φ_Clk_ = Φ_2_ = Φ_3_ = 180°). The DI between the incident beams causes ‘0’ output to occur at P_out_ (i.e., T < T_th_) when the combination (11), along with the Clk at various phases (i.e., Φ_Clk_ = 180°, Φ_2_ = 0°, and Φ_3_ = 90°), is launched. In this manner, the XOR logic gate is formed. Figure 6 depicts the field intensity distributions of the XOR gate propagating through the Z-shaped silicon waveguide at 1.55 μm.

Our waveguide can attain a high CR = 29 dB at 1.55 μm due to the relative difference between $P_{\mathrm{mean}}^{1}\;$and $P_{\mathrm{mean}}^{0}.$ Table 2 provides an overview of the XOR simulation outcomes.

### 4.2. AND

**Figure 7:**
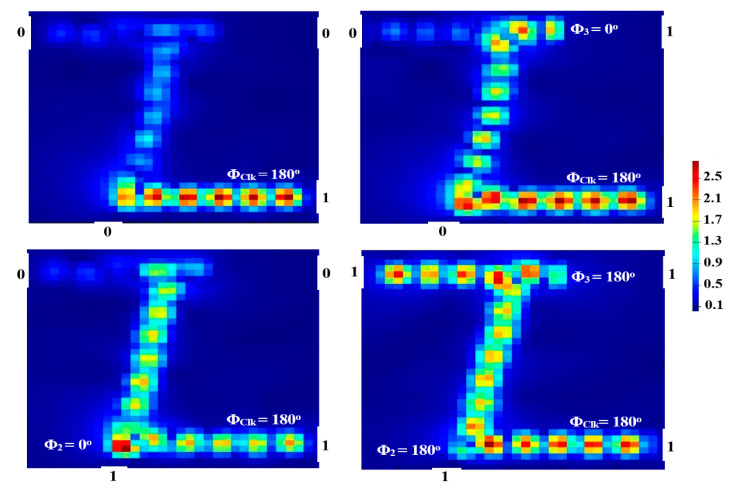
AND field intensity distributions propagating through Z-shaped silicon waveguide at 1.55 μm.

When all incident beams enter the suggested waveguide at the same phase (i.e., Φ_Clk_ = Φ_2_ = Φ_3_ = 180°), P_out_ yields ‘1’ as a result of CI. When these incident beams are injected at a different phase, P_out_ emits ‘0’. In this way, the output is ‘1’ only when all inputs are ‘1’, which corresponds to the AND gate, as seen in Figure 7.

Our waveguide results in a significant CR = 33.26 dB at 1.55 μm. Table 3 lists the rest of the outcomes of the AND simulation.

### 4.3. OR

**Figure 8:**
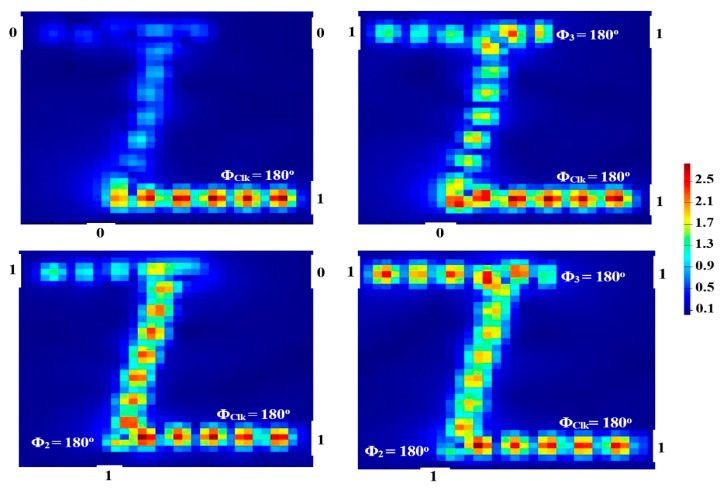
OR field intensity distributions propagating through Z-shaped silicon waveguide at 1.55 μm.

When the Clk is entered with the combination of input beams (01, 10, or 11) at the same phase of 180°, the result of P_out_ is ‘1’. The OR is thus realized as shown in Figure 8.

The OR outcomes at 1.55 μm are displayed in Table 4 in terms of T and CR. A high CR = 31.51 dB is achieved because of the significant disparity between $P_{\mathrm{mean}}^{1}\;$and $P_{\mathrm{mean}}^{0}$.

The XOR, AND, and OR gates depend on the Clk beam to operate properly. Therefore, we evaluate how well each of these three operations performs at 1.55 μm in the presence of the Clk (i.e., meaning P_in1_ has ‘1’ input) and in the absence of the Clk (i.e., meaning P_in1_ has ‘0’ input) using the suggested waveguide. Table 5 shows that using the Clk in the waveguide-based scheme results in much higher CRs than without using it.

## 5. NOT, NOR, NAND, XNOR

To execute the inverted logic gates, a Clk with an angle of 0° must be sent from P_in3_ of Figure 1.

### 5.1. NOT

**Figure 9:**
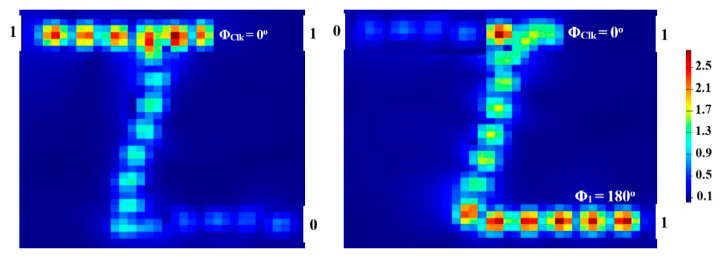
NOT field intensity distributions propagating through Z-shaped silicon waveguide at 1.55 μm.

One beam is inserted into P_in1_ at a different phase of 180° to implement the NOT gate. When P_in1_ is set to ‘1’, P_out_ generates a logical ‘0’ (i.e., T < T_th_) due to the DI that happens as a result of the input beams’ various phase conditions. When P_in1_ is set to ‘0’, the Clk (all ‘1’s) does not undergo a differential phase and generates instead a logical ‘1’ (i.e., T > T_th_) at P_out_. This results in the implementation of the NOT gate, as displayed in Figure 9.

$P_{\mathrm{mean}}^{1}\;$and $P_{\mathrm{mean}}^{0}$ have a significant discrepancy, which results in a high CR of 31.76 dB. Table 6 lists the NOT outcomes utilizing the suggested waveguide at 1.55 μm.

### 5.2. NOR

**Figure 10:**
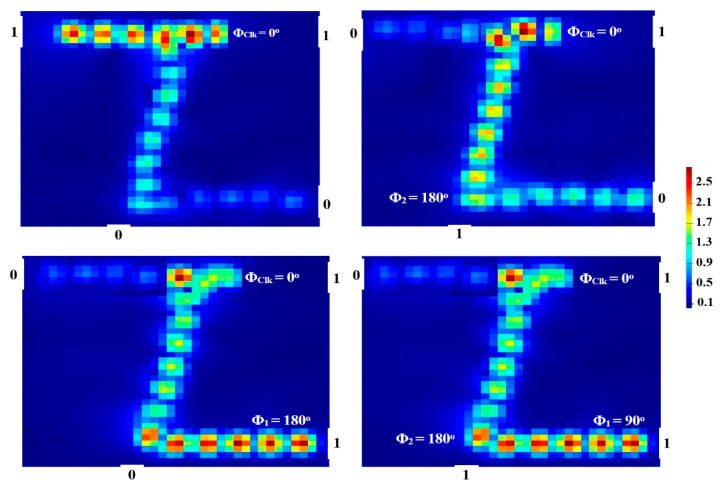
NOR field intensity distributions propagating through Z-shaped silicon waveguide at 1.55 μm.

Two beams are injected into P_in1_ and P_in2_ of Figure 1 to carry out the NOR (NOT-OR) operation. When (01, 10, or 11) are combined and injected at different angles, DI results in a logical ‘0’ at P_out_. If (00) is launched, the Clk with Φ_Clk_ = 0° will negate the phase balance of the three ports, resulting in ‘1’ at P_out_. This leads to the realization of the NOR function, as seen in Figure 10.

Our waveguide achieves a high CR = 31.50 dB for the NOR gate, as shown in Table 7.

### 5.3. NAND

**Figure 11:**
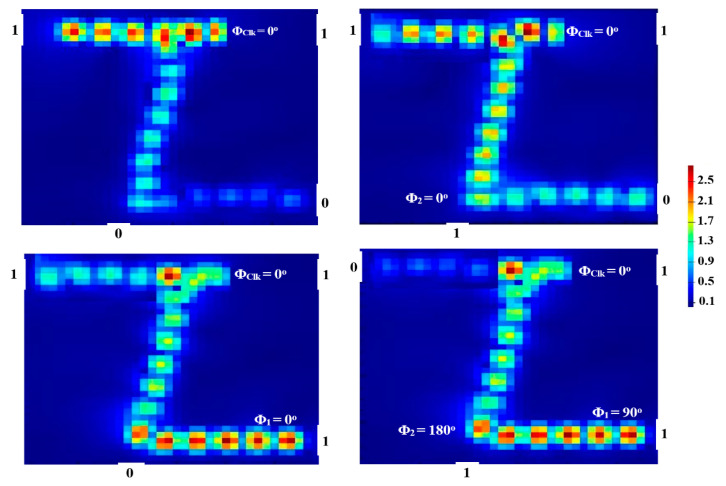
NAND field intensity distributions propagating through Z-shaped silicon waveguide at 1.55 μm.

The NAND (NOT-AND) gate can be realized by injecting the Clk into P_in3_ and the other two beams into P_in1_ and P_in2_ so that the output is ‘0’ if and only if all inputs are ‘1’. When P_in1_ and P_in2_ are both “OFF” (i.e., 00), the Clk having a Φ_Clk_ = 0° causes the output to become ‘1’. CI simply occurs when the Clk and (01, 10) are launched at the same angle of 0°, resulting in a ‘1’ output. The concomitant DI causes a ‘0’ output when the logic combination (11) is launched with the Clk at Φ_1_ = 90°, Φ_2_ = 180°, and Φ_Clk_ = 0° as shown in Figure 11.

$P_{\mathrm{mean}}^{1}$ is higher than$\;P_{\mathrm{mean}}^{0}$, thus leading to a high CR = 29.83 dB. The numerical outcomes of the NAND are cited in Table 8.

### 5.4. XNOR

**Figure 12:**
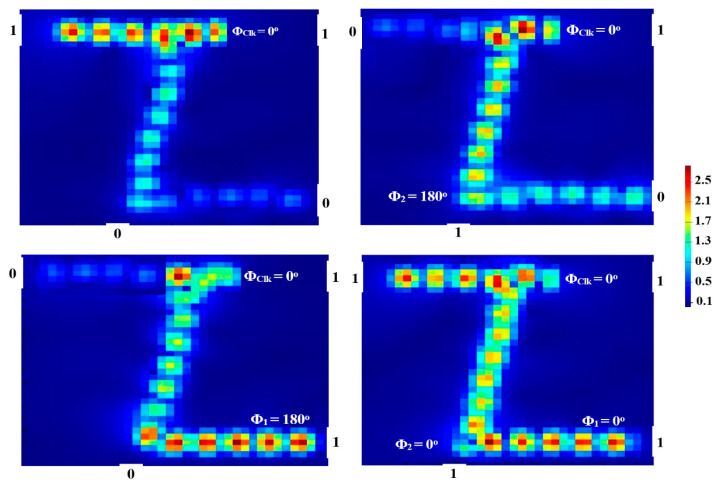
XNOR field intensity distributions propagating through Z-shaped silicon waveguide at 1.55 μm.

The Clk enters P_in3_ to build the XNOR (exclusive-XOR) gate, similar to NOR and NAND gates, while the other two beams are injected from P_in1_ and P_in2_. When the input beams are combined (11) and the Clk is inserted at 0°, P_out_ emits a ‘1’ as a result of CI. When (01) or (10) is launched with a different phase, on the other hand, P_out_ generates a ‘0’, as depicted in Figure 12.

The XNOR has a high CR of 31.33 dB due to the huge discrepancy between $P_{\mathrm{mean}}^{1}\;$and $P_{\mathrm{mean}}^{0}$. Table 9 lists the XNOR outcomes.

## 6. Comparison

Table 10 compares the ability of the suggested waveguide to realize AOLGs to those reported in the literature and used for the same purpose in terms of the building platform, operating wavelength, and achieved CR. The data shown in this table provide evidence that our waveguide is able to carry out the required logic operations with considerably greater performance in a way that is both technically and commercially feasible.

The limitations on manufacturing are frequently referred to as a bottleneck. Nanophotonics devices are used in more applications, which makes the photonic design more difficult and complex. Instead of using conventional photonic design procedures to address this issue, designers are increasingly turning to cutting-edge optimization techniques [43,44,45,46]. In contrast to the traditional methods, which involve changing relatively simple known geometries with a limited number of parameters, these new methodologies evaluate devices with totally arbitrary geometries. Devices have been developed with extraordinarily small footprints, excellent efficiency, and novel characteristics that cannot be achieved using conventional methods in order to make use of the additional degrees of freedom [47,48,49,50,51,52,53,54,55]. Silicon and silica, which are abundant in the earth’s crust and significant elements of the earth’s mantle, make up the proposed waveguide. Due to the availability of 3D FLDW technology [33,34,35,36,37,38] and lithographic manufacturing processes [23,39,40,41], it is, therefore, possible to anticipate the experimental verification of the proposed waveguide based on the major findings of this simulation. Instead of being an essential barrier, this is a technological matter issue that can be solved in practice. Additionally, the experimental implementation of multiple AOLGs based on various optical waveguides has been reported in recent years [19,23,24,56,57], paving the way to similar implementations.

## 7. Conclusions

Using appropriately driven Z-shaped silicon-on-silica waveguides, a set of basic logic gates were simulated at 1.55 μm. These logic gates operate according to CI and DI, which manifests as a result of the phase difference experienced by the launched input optical beams. FDTD solutions in Lumerical, the commercially available software, were used to simulate the target logic gates. The CR metric was employed to evaluate how well these logic gates perform. The impact of key operating parameters on the waveguide performance was investigated and assessed. The simulation results have demonstrated that the proposed waveguide can achieve higher CRs and speed compared to other reported designs. The proposed single AOLGs hold the promise of being connected and combined to form more sophisticated digital circuits of enhanced functionality and multistage information processing architectures. This estimate is based both on their principle of operation and technological feasibility. To this end, issues such as fan-in/fan-out capability, power consumption, tolerable attenuation, fabrication platform, building complexity, and overall practicality should be taken into account in order to identify possible trade-offs and derive specific design rules that will render this possible in a performance- and cost-efficient manner.

## Figures and Tables

**Table 1:** Default parameters.

Symbol	Definition	Value	Unit
L_1_	Length of short slot	1.0	μm
L_2_	Length of long slot	1.5	μm
w	Width of slot	0.3	μm
d	Thickness of slot	0.3	μm
θ	Angle between slots	70	degree
λ	Operating wavelength	1.55	μm
T_th_	Threshold transmission	0.12	-

**Table 2:** XOR outcomes (T_th_ = 0.12).

P_in1_ (Clk)	P_in2_	P_in3_	T	P_out_	CR (dB)
1	0	0	0.028	0	29
1	0	1	0.576	1	
1	1	0	0.552	1	
1	1	1	0.034	0	

**Table 3:** AND outcomes (T_th_ = 0.12).

P_in1_ (Clk)	P_in2_	P_in3_	T	P_out_	CR (dB)
1	0	0	0.028	0	33.26
1	0	1	0.036	0	
1	1	0	0.026	0	
1	1	1	0.835	1	

**Table 4:** OR outcomes (T_th_ = 0.12).

P_in1_ (Clk)	P_in2_	P_in3_	T	P_out_	CR (dB)
1	0	0	0.028	0	31.51
1	0	1	0.576	1	
1	1	0	0.552	1	
1	1	1	0.835	1	

**Table 5:** Comparison of CR with and without Clk beam.

Gate	CR (dB) with Clk	CR (dB) without Clk
XOR	29	8.2
AND	33.26	10.6
OR	31.51	9.8

**Table 6:** NOT outcomes (T_th_ = 0.12).

P_in1_	P_in3_ (Clk)	T	P_out_	CR (dB)
0	1	0.862	0	31.76
1	1	0.036	1	

**Table 7:** NOR outcomes (T_th_ = 0.12).

P_in1_	P_in2_	P_in3_ (Clk)	T	P_out_	CR (dB)
0	0	1	0.862	1	31.50
0	1	1	0.038	0	
1	0	1	0.036	0	
1	1	1	0.036	0	

**Table 8:** NAND outcomes (T_th_ = 0.12).

P_in1_	P_in2_	P_in3_ (Clk)	T	P_out_	CR (dB)
0	0	1	0.862	1	29.83
0	1	1	0.735	1	
1	0	1	0.536	1	
1	1	1	0.036	0	

**Table 9:** XNOR outcomes (T_th_ = 0.12).

P_in1_	P_in2_	P_in3_ (Clk)	T	P_out_	CR (dB)
0	0	1	0.862	1	31.33
0	1	1	0.038	0	
1	0	1	0.036	0	
1	1	1	0.836	1	

**Table 10:** Comparison of proposed and other waveguides-based AOLGs in terms of building platform, size, operating wavelength, and achieved CR.

Gates	Platform	Size	Wavelength (nm)	CR (dB)	Refs.
AND, XOR, OR, NOT, NAND, NOR XNOR	PC waveguides	-	1550	5.42–9.59	[8]
AND, XOR, OR	T-shaped PC waveguides	9 μm × 5 μm	1550	8.29–33.05	[12,13,14]
AND, NOR, XNOR	Silicon photonics platform	3 μm × 1.5 μm	1550	>10 dB	[19]
XOR, AND, OR, NOT, NOR, XNOR, NAND	Silicon-on-silica waveguides	1.5 μm × 2.36 μm	1550	20.51–30.33	[22]
NOT, XOR, AND, OR, NOR, NAND, XNOR	Metal slot waveguide	5.33 μm × 0.42 μm	632.8	6–16	[24]
NOT, XOR, AND, OR, NOR, NAND, XNOR	Metal-insulator-metal structures	50 μm × 2 μm	632.8	15	[25]
NOT, XOR, AND, OR, NOR, NAND, XNOR	Dielectric-metal-dielectric design	0.4 μm × 0.15 μm	900 and 1330	5.37–22	[26]
XOR, AND, OR, NOR, NAND, XNOR	Dielectric-loaded waveguides	-	471	24.41–33.39	[27]
XOR, AND, OR, NOT, NOR, XNOR, NAND	Z-shaped silicon waveguides	1.0 μm × 1.5 μm	1550	29–33.26	This work

## Data Availability

Not applicable.
